# The Presence of Potentially Pathogenic Protozoa in Lettuce (*Lactuca sativa*) Sold in Markets in the Central Peruvian Andes

**DOI:** 10.3390/ijerph20020943

**Published:** 2023-01-04

**Authors:** J. Raul Lucas, Daphne Ramos, S. Sonia Balcázar, Carlos Santos

**Affiliations:** 1 Department of Animal Health and Public Health, Veterinary Medicine Faculty, Universidad Nacional Mayor de San Marcos, San Borja, Lima 15021, Peru; 2 Department Section of Food Technology, Veterinary Faculty, Complutense University, 28040 Madrid, Spain

**Keywords:** *Balantidium coli*, *Cryptosporidium*, protozoa, parasite, foodborne disease, lettuce

## Abstract

Peru is currently one of the world’s leading culinary destinations, whose world-renowned cuisine uses vegetables, mainly lettuce, as frequent ingredients. Vegetable consumption is promoted worldwide as a part of a healthy diet. However, vegetables, more frequently lettuce, have been implicated as a vehicle of infection for several foodborne parasites. This study aimed to determine the presence of potentially pathogenic parasites in lettuce marketed in the Central Andes of Peru. A total of 75 lettuce samples were collected from the two largest wholesale markets and the main open-air market in Jauja province, in the central Peruvian Andes. The province of provenance (coast vs. highlands), lettuce variety (“curly-leaf”, “iceberg”, and “butter”), and type of market were recorded. The samples were microscopically examined for detection of parasitic life forms using standard parasitological methods including direct slide smear, Lugol’s iodine staining, and Modified Ziehl–Neelsen staining. The overall positivity of parasitic contamination in lettuces was 45.3% (CI 95%: 34–56.6%). *Cryptosporidium* spp., *Isospora belli*, *Giardia lamblia*, *Balantidium coli*, and *Entamoeba* spp. were detected in twenty-six (34.7%), six (8%), four (5.3%), two (2.7%), and two (2.7%) lettuces, respectively. *I. belli* was found in a significantly (*p* < 0.01) lower proportion in the “butter” variety, and significantly (*p* < 0.05) higher contamination with *G. lamblia* was found in lettuce sold at the open-air market. *B. coli*, *G. lamblia*, and *E. histolytica/E. dispar/E. moshkovskii* were detected only in lettuce from the highlands (Tarma province). This study provides important data for health authorities to develop food safety programs. This information is also of interest to the international community because of the increased visibility that Peru has gained as a tourist destination.

## 1. Introduction

Over the past decades, scientific research has consistently confirmed that fruit and vegetable consumption is an essential component of a healthy diet, as it helps prevent chronic diseases such as cardiovascular disease and certain cancers [1]. An estimated 3.9 million deaths worldwide were associated with poor fruit and vegetable consumption in 2017 [1].

However, vegetable salads are prepared and consumed without any heat treatment to preserve their taste and content of heat-labile nutrients, and they have been recognized as a vehicle of infection for several undesirable microorganisms that are often present at the time of sale to the consumer [2,3]. In developing countries, foodborne parasite infections are often endemic because of increased contamination of fresh produce as a result of inadequate sanitation systems [4].

Moreover, globalization facilitates the spread of foodborne parasitic diseases, which can have enormous negative sanitary and economic effects. This is not only a consequence of the increased movement of food products but also of the movement of people. Immigrants, business travelers, and tourists have the potential to contract and spread foodborne parasitic infections acquired in developing countries [3].

Peru is a developing country that has experienced a gastronomic and tourism boom in recent decades. To date, Peru has been the winner of the “World’s Leading Culinary Destination” award since it was established in 2012, with the exception of 2019, and has been the only winner of the “South America’s Leading Culinary Destination” award since it was first instituted. Other awards such as “World’s Leading Cultural Destination” or “World’s Leading Tourist Attraction” have also been granted to this country [5]. Also, this country has emerged as one of the fastest-growing stable economies in South America [6].

However, this remarkable economic growth contrasts with the country’s public health deficiencies. Public health surveillance in Peru has been inadequately addressed, especially with regard to foodborne diseases. Peru is one of six countries in the Americas categorized in WHO subregion stratum “D”, i.e., there is high infant and adult mortality as a result of foodborne diseases [7]. One of the main factors behind this problem is the use of contaminated water for the irrigation of fruits and vegetables, which can be a source of infectious diseases [8].

Among all the vegetables eaten raw in Peruvian cuisine, lettuce (*Lactuca sativa*) is probably the most common. Renowned Peruvian dishes such as “ceviche” (marinated fish), “papa a la huancaína” (Huancayo-style potatoes), “pollo a la brasa” (roast chicken), “causa” (mashed yellow potato dumpling), and many others use lettuce as an edible garnish or as an essential part of the salad that is served as a side dish. Since lettuce grows in contact with soil during its cultivation, it can be easily contaminated by several parasites, mainly protozoa, through irrigation water, as has been reported worldwide [9,10,11,12,13,14,15].

*Giardia* and *Cryptosporidium* are foodborne parasites causing significant health and economic impacts of particular concern [16,17]. Other pathogenic protozoa, such as *Balantidium coli*, *Entamoeba histolytica*, or *Isospora belli*, have gained attention for causing infections that have the potential to trigger foodborne disease outbreaks [18,19,20,21].

The types of parasites contaminating vegetables and their prevalence are influenced by climatic, ecological, and human factors [22]. Therefore, local data on contamination status helps health authorities to develop regional food safety programs by implementing specific control measures. Although it is essential to know the prevalence in fresh produce, baseline studies are scarce throughout the Peruvian food system. Peru is currently one of the world’s leading tourist and culinary destinations, which makes this information also important to the international community. Consequently, this study aimed to determine the presence of potentially pathogenic parasites in lettuce marketed in the Central Andes of Peru.

## 2. Material and Methods

### 2.1. Study Area and Samples

**Figure 1:**
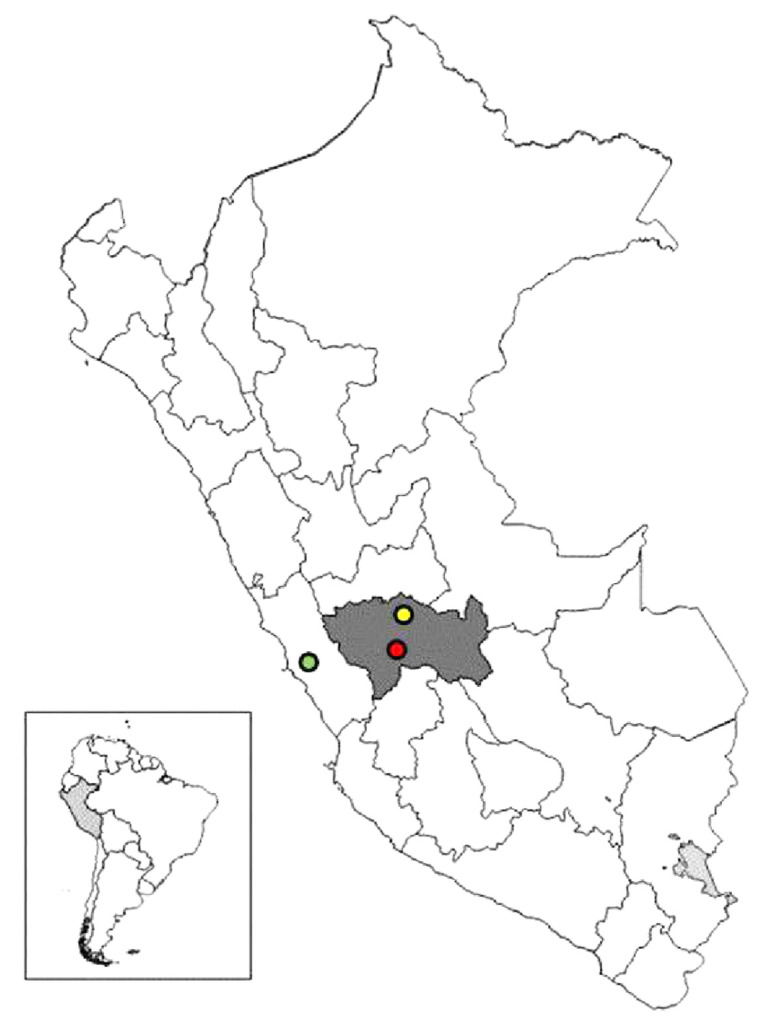
Location of Jauja province (●) and Junin Region (dark gray), Central Andes of Peru, South America. Lima province (●) and Tarma province (●) are also shown.

This study analyzed 75 samples of fresh lettuce obtained from the two largest wholesale markets (“Mayorista” and “Modelo”) and the main open-air market (“Feria”) in Jauja Province, Junin Region, in the central Peruvian Andes (altitude 3400 m, latitude 11°46′ S, longitude 75°30′ W). This is the second most populated province in the region and is endemic for many parasitic zoonoses, such as fasciolosis, echinococcosis, sarcocystosis, cryptosporidiosis, and other zoonotic protozoan diseases [23,24,25]. In this agricultural region, daily temperatures range from 0 to 23 °C and the climate is dry, with two distinct seasons: a rainy season from November to April and a dry season from May to October. Three types or varieties of lettuce are available in the province’s markets: curly-leaf (Waldman Green), iceberg (Great Lakes), and butter (Dark Green Boston), produced in Tarma Province (Junin, Andean region or highlands at about 3000 m above sea level) and Lima (coastal region at 0–160 m above sea level) (Figure 1).

### 2.2. Sample Collection

Four vegetable stalls from each of the three markets were randomly selected each week, between February and March 2016. From each selected stall, one to three lettuces were randomly selected (one of each type). Samples were individually collected into sterile, labeled, plastic bags and immediately transported at 4 °C in a cold box to the Instituto de Investigaciones Veterinarias Tropicales y de Altura (IVITA) of the Universidad Nacional Mayor de San Marcos for parasitological analysis. The provenance of the lettuces was obtained from the sellers’ records.

### 2.3. Sample Processing

A protocol commonly used for the detection of parasites on vegetables was used, involving a first washing or extraction step, followed by concentration and subsequent identification, as described elsewhere [26]. Briefly, shallow, wilted, stained, or damaged leaves of these vegetables were removed and not used in the analysis. The intact part of each lettuce (approximately 250 g) was separated leaf by leaf. These leaves were washed individually using a wash bottle with 0.85% NaCl. The resulting liquid was filtered through sterile gauze and transferred to a conical flask. Overnight (approximately 12 h) sedimentation of this washing solution was performed. The supernatant from each sample was discarded, and the sediment was resuspended with 15 mL of 0.85% NaCl and centrifuged at 3000 rpm for 5 min. After discarding the supernatant, stained and unstained (direct) smears were prepared from the sediment. For the direct smear, a drop of the sediment (previously homogenized by shaking) was applied onto a clean slide, and a cover slip was placed. Iodine-stained smears were performed by adding a drop of Lugol’s iodine before the coverslip was placed. Sediment was also processed by a modified Ziehl–Neelsen staining technique for the identification of oocysts of *Cryptosporidium*, as described elsewhere [15,27]. Fifteen slides were prepared for each sample, including five unstained, five stained with Lugol’s iodine, and five stained with Ziehl–Neelsen.

### 2.4. Parasite Detection

Slides were examined under an optical microscope (Carl Zeiss) with a digital camera (AxionCam ERc5s, Carl Zeiss) and ZEN 2012 SP1 measurement software (Blue edition, Carl Zeiss). It was used for the 10×, 40×, and 100× objectives for the analysis. Infective parasitic forms were identified based on the study of their morphological characteristics and their corresponding measurements. *Giardia lamblia* cysts are oval-shaped, double-walled, 8 to 14 µm long, and 7 to 10 µm wide, and they contain four nuclei at a pole of the cyst and filaments [22]. The sporulated oocyst of *Isospora belli* was determined based on the size, 23–36 × 8–17 µm, the presence of two sporocysts, and, above all, by its ellipsoidal shape; in fact, only oocysts with a shape index greater than 1.2 were considered [18,28]. Parasitological diagnosis for *Balantidium coli* was based on the observation of the oval-shaped and cilia-covered trophozoite, with a length of 30–150 µm and a width of 25–120 µm. The *B. coli* cyst can be spherical or slightly ovoid and has a diameter of 40–60 µm. The macronucleus of this species is sausage-shaped, and the micronucleus is smaller and globular [19,22]. *Entamoeba* spp. (*E. histolytica*/*E. dispar*/*E. moshkovskii*) cysts were identified by their spherical shape, size of 10–15 µm, and four nuclei with central nucleoli [19]. Oocysts of *Cryptosporidium* spp. measuring between 4 and 6 µm in diameter were considered positive and should be visualized as spherical, red- to fuchsia-colored with dark granulations inside [19].

### 2.5. Statistical Analysis

The Chi-square test was used to assess differences in the prevalence of contamination between lettuce varieties, their provenance, and the types of markets. The data was analyzed using the SPSS v.25.0 (IBM Corp., Armonk, NY, USA) statistical software. A *p*-value ≤ 0.05 was considered to be statistically significant.

## 3. Results

**Figure 2:**
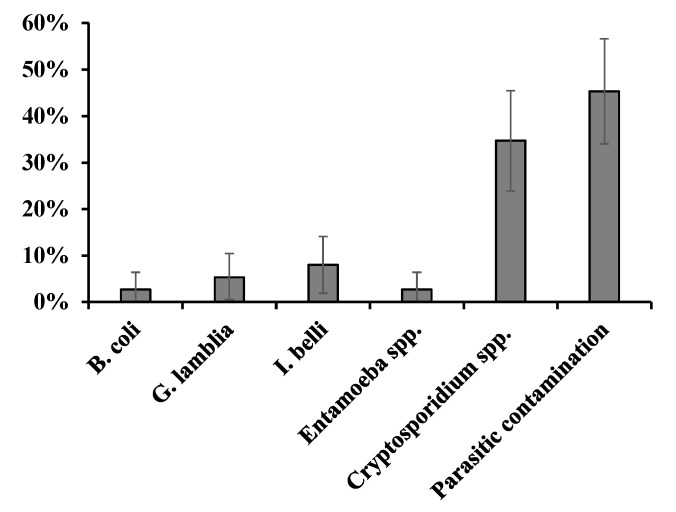
Prevalence of pathogenic parasites in fresh lettuce marketed in Jauja province in the central Peruvian Andes.

**Figure 3:**
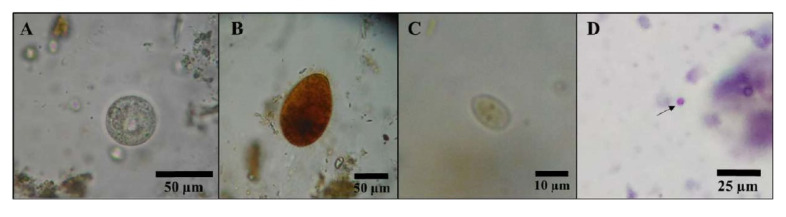
Some parasites found in this survey: (**A**) *Balantidium coli* cyst. (**B**) *Balantidium coli* trophozoite. (**C**) *Giardia lamblia* cyst. (**D**) Oocysts of *Cryptosporidium* spp. (arrow) stained by the modified Ziehl–Neelsen technique.

Figure 2 shows the prevalence of pathogenic parasites in fresh lettuce marketed in the central Peruvian Andes (Jauja). 45.3% (CI 95%: 34–56.6%) of the lettuce evaluated had at least one potentially pathogenic parasite. Figure 3 shows some of these parasites. The most frequently detected parasites were *Cryptosporidium* spp. (34.7%, CI 95%: 23.9–45.5%), followed by *I. belli* (8%, CI 95%: 1.9–14.1%). *Giardia lamblia*, *B. coli*, and *Entamoeba* spp. were detected to a lesser extent, in four (5.3%), two (2.7%), and two (2.7%) lettuces, respectively.

Table 1 shows the influence of lettuce variety, market type, and origin of lettuce on the prevalence of the parasites identified in these vegetables. *I. belli* was found in a significantly (*p* < 0.01) lower proportion in the Boston Dark Green (“butter”) variety. A significantly higher (*p* < 0.05) contamination with *G. lamblia* was found in lettuce sold at the main open-air market (“Feria”). *B. coli*, *G. lamblia*, and *E. histolytica/E. dispar/E. moshkovskii* were detected only in lettuce from Tarma.

## 4. Discussion

Foodborne parasitic diseases are among the most important neglected infectious diseases in the world. This study shows that the prevalence of pathogenic parasites in fresh lettuce marketed in the central Peruvian Andes is similar to that reported by other authors using similar parasitological methods. For instance, in Ghana, 61% of the lettuces analyzed showed parasitic contamination, mainly by the presence of *Cryptosporidium* (17.6%), *E. hystolitica* (5.8%), and *G. lamblia* (4.9%) [9]. In Egypt, lettuce showed *G. lamblia* cysts (16%) and *Entamoeba* spp. (13.9%) contamination [10]. Gabre and Shakir [11] reported parasitic contamination of 13.04% of lettuce sold in Saudi Arabia, mainly reporting *Entamoeba* spp. Similarly in Sudan, 36.4% of marketed lettuce was contaminated, showing a prevalence of 18.2% and 9% for *Entamoeba* spp. and *G. lamblia*, respectively [12]. In Cameroon, lettuces showed a contamination prevalence of approximately 30% for both *Entamoeba* sp. and *B. coli* [13]. Machado et al. [14] reported 100% parasitic contamination of lettuce sampled in Brazil, with a high level of detection of *B. coli*. In Ethiopia, in a study evaluating 23 lettuces, *Cryptosporidium* spp. and *B. coli* were detected in 8.7% and 4%, respectively [15].

In this study, the most frequently identified parasite was *Cryptosporidium* spp. (34.7%, CI 95%: 23.9–45.5%). Light microscopy is not able to determine the species of this parasite, and further molecular studies would be required, which were not performed in the present study due to economic and logistical limitations. However, although the species most frequently affecting humans are *Cryptosporidium hominis* and *Cryptosporidium parvum*, at least 19 species (out of 41 described) have been reported to be capable of infecting humans [29], causing cryptosporidiosis, a disease with significant health and economic impact [17]. In the United States, it was estimated that about 8% of *Cryptosporidium* infections are foodborne [30]. Moreover, vegetable salads have been the most frequent food contaminated in foodborne outbreaks of cryptosporidiosis since 1984 [31].

In this survey, *Isospora belli* was found in 8% (CI 95%: 1.9–14.1%) of the lettuces. *I. belli* is a coccidian protozoan that causes human isosporiasis, coccidiosis, or traveler’s disease. This infection is usually asymptomatic, so the prevalence is often underestimated. However, isosporiasis is relevant in immunocompromised people, especially AIDS patients. Water and food contaminated with *I. belli* oocysts are the main routes of infection for humans [18,19].

In the present study, *Giardia lamblia, B. coli*, and *Entamoeba* spp. have shown the lowest prevalence, 5.3%, 2.7%, and 2.7%, respectively. Human ingestion of *G. lamblia* cysts causes a neglected disease known as giardiasis. This is an important public health problem with a serious social and economic burden worldwide [16]. Giardiasis, also known as traveler’s diarrhea, is characterized by watery diarrhea and is associated with places with inadequate sanitary practices [20].

*B. coli* is the only known ciliate protozoan of zoonotic significance, as it causes balantidial dysentery (balantidiasis) in humans. Balantidiasis has a worldwide distribution, although the highest rates are observed in tropical and sub-tropical areas [21]. Balantidiasis can be fatal, but most *B. coli* infections are asymptomatic, and therefore it is a neglected disease that is not considered to be of public health importance [32]. In Peru, in the southern Andean region, there is a reported prevalence of *B. coli* of 6.6% and 5% in children and adults, respectively [33]. Also, a fatal case of colonic balantidiasis has been reported in a livestock farmer from the Peruvian Andes [34].

The primary *B. coli* reservoir is the pig, which usually contaminates the environment through its feces. Transmission of the parasite occurs via the fecal–oral route, i.e., the host is infected by ingesting food and water contaminated with cysts [21]. It is assumed that trophozoites cannot survive in stomach conditions [35]. However, research is still needed to clarify the trophozoites’ role as an infectious stage [21].

*Entamoeba histolytica* is a pathogenic parasite responsible for amoebic dysentery. Humans are the major reservoir of the organism and, under unsanitary living conditions or unhygienic food preparation practices, can contaminate water and food [20]. *E. dispar* and *E. moshkovskii* are commensal, non-pathogenic parasites that are morphologically identical to *E. histolytica*. For this reason, the most accurate and recommended designation is *E. histolytica*/*E. dispar*/*E. moshkovskii* [19,36].

In the present work, *I. belli* was found in a significantly (*p* < 0.01) lower proportion in the Boston Dark Green variety. Similarly, Machado et al. [14] reported significant differences in the presence and level of contamination of potentially pathogenic parasites, such as *B. coli*, *Entamoeba* sp., *Toxocara* sp., *Trichuris* sp., among others, depending on the lettuce variety (curly, red or looseleaf). A significant difference in the presence of parasites has also been described depending on the type of cropping system applied in lettuce cultivation (traditional, organic, and hydroponic) [37].

A significantly higher (*p* < 0.05) contamination with *G. lamblia* was found in lettuce sold at the main open-air market (“Feria”). In this type of market, Peruvian public health authorities have less control over the conditions of the stalls, i.e., food would be sold at ground level, and often different products (e.g., vegetables and meat) are offered in the same place. Further studies are needed to determine whether these factors may contribute to parasitic contamination. Muñoz et al. [2] described factors such as poor display conditions, lack of concrete floors, and inadequate market stall layout as related to an increase in the contamination of vegetables with pathogenic microorganisms.

Overall, these results show that lettuce marketed in this Andean region is a potential source for the spread of various parasitic diseases and therefore poses a significant risk if consumed after being improperly washed. Worldwide, lettuce is one of the most dangerous vegetables because of the presence of pathogens. It was determined that the risk of contamination was 65 times higher in lettuce samples than in tarragon samples and more than 7, 9, and 30 times higher than in spearmint, watercress, and coriander samples, respectively [38]. Therefore, surveillance of parasitic contamination in raw vegetables used in salads, such as lettuce, is a crucial measure to control the occurrence of human gastroenterological diseases [38].

In Peru, and especially in the Andean region, the prevalence of parasites such as *Giardia lamblia*, *Entamoeba* spp., or *Cryptosporidium* spp. has been described in apparently healthy and hospitalized children [39,40,41,42]. However, gastroenterological diseases caused by protozoa are often unnoticed and underestimated worldwide, resulting in their current neglect, despite the mortality and disability-adjusted life years (DALYs) they entail [43]. For example, *Giardia lamblia*, *Entamoeba* spp., and *Cryptosporidium* spp. have caused worldwide more than 183, 103, and 64 million cases, respectively, 0.2, 0.5, and 2 million DALYs, respectively, and in total more than 33,000 deaths [43].

In addition, parasitic contamination of vegetables can be considered an indicator of deficiency in good agricultural practices, as it has been commonly associated with factors such as inadequate hygiene, poor sanitary facilities, the use of untreated manure as fertilizer, and the use of wastewater in vegetable cultivation [4,8]. Peru is one of the most popular culinary destinations in the world. However, efforts to improve public health surveillance are insufficient, and food safety receives relatively little political attention in this country, especially in the Andean region. In this scenario, local surveys are essential to attract consumer attention, mobilize the necessary resources, and establish food safety as a public health priority.

## 5. Conclusions

The presence of parasites such as *Cryptosporidium* spp., *Isospora belli*, *Giardia lamblia*, *Balantidium coli*, and *Entamoeba* spp. in lettuce sold in the central Andes of Peru, poses a health risk to local consumers and foreign visitors.

## Figures and Tables

**Table 1:** Prevalence of parasites in three varieties of lettuce (*Lactuca sativa*) from two Peruvian provinces (Tarma and Lima) and commercialized in three types of markets in Jauja province, in the central Peruvian Andes.

Parasite	No. (%) Positive							
	Lettuce Variety			Type of Market			Province of Origin	
	Butter	Curly-Leaf	Iceberg	“Modelo”	“Mayorista”	“Feria”	Tarma	Lima
	(*n* = 40)	(*n* = 20)	(*n* = 15)	(*n* = 25)	(*n* = 25)	(*n* = 25)	(*n* = 60)	(*n* = 15)
*Balantidium coli*	1 (2.5)	1 (5)	0 (0)	1 (4)	0(0)	1 (4)	2 (3.3)	0 (0)
*Giardia lamblia*	1 (2.5)	3 (15)	0 (0)	0 (0)	0 (0)	4 (16) **	4 (6.7)	0 (0)
*Isospora belli*	0 (0) *	4 (20)	2 (13.3)	3 (12)	1 (4)	2 (8)	4 (6.7)	2 (13.3)
*E. histolytica*/*E. dispar*/*E. moshkovskii*	1 (2.5)	1 (5)	0 (0)	0 (0)	0 (0)	2 (8)	2 (3.3)	0 (0)
*Cryptosporidium* spp.	14 (35)	5 (25)	7 (46.7)	11 (44)	10 (40)	5 (20)	19 (31.7)	7 (46.7)
Parasitic contamination	17 (42.5)	9 (45)	8 (53.3)	14 (56)	10 (40)	10 (40)	26 (43.3)	8 (53.3)

* *Isospora belli* cysts were detected significantly more often in curly-leaf and iceberg lettuce than butter variety (*p* < 0.01). ** *Giardia lamblia* cysts were detected significantly more often in open-air market (“Feria”) than wholesale markets (*p* < 0.05).
